# Relationship Between Body Composition Parameters and Metabolic Syndrome in Young Thai Adults

**DOI:** 10.4274/jcrpe.1576

**Published:** 2014-12-05

**Authors:** Sirianong Namwongprom, Kittipan Rerkasem, Antika Wongthanee, Sakda Pruenglampoo, Ampica Mangklabruks

**Affiliations:** 1 Chiang Mai University Faculty of Medicine, Department of Radiology, Chiang Mai, Thailand; 2 Chiang Mai University Faculty of Medicine, Clinical Epidemiology Unit, Chiang Mai, Thailand; 3 Chiang Mai University Faculty of Medicine, Department of Surgery, Chiang Mai, Thailand; 4 Chiang Mai University The Research Institute for Health Sciences, Chiang Mai, Thailand; 5 Chiang Mai University Faculty of Medicine, Department of Internal Medicine, Chiang Mai, Thailand

**Keywords:** metabolic syndrome, android fat mass, gynoid fat mass, android to gynoid fat mass ratio, dual-energy x-ray absorptiometry

## Abstract

**Objective:** The aim of this study was to evaluate the relationship between body composition parameters, i.e. waist circumference, android fat mass (AFM), gynoid fat mass (GFM), android to gynoid fat mass ratio (AG ratio) and metabolic syndrome (MS) risk components in young Thai adults.

**Methods:** This was a cross-sectional study conducted among 391 adolescents (174 male, 217 female). The body mass index (BMI), waist circumference, blood pressure, triglyceride, high-density lipoprotein (HDL) cholesterol and glucose levels were determined. AFM, GFM and AG ratio were assessed by dual-energy X-ray absorptiometry (DXA). Linear regression analysis was done to assess the relationship of waist circumference, AFM, GFM and AG ratio with MS risk components’ score, separately.

**Results:** Among 391 young adults aged 18.5-21.8 years, MS was found in 5.9%. Participants with MS (n=23) had a significantly higher weight, height and BMI than those without MS. There was no statistically significant difference in bone mineral density between the two groups. At univariable linear regression analysis, waist circumferences, AFM, GFM and AG ratio showed significant relationship with MS risk components’ score. However, after adjusting for gender, birth weight and BMI, AG ratio demonstrated greater relationship with MS risk components’ score (β 1.89, 95%CI 1.096-2.978) than waist circumference (β 0.046, 95%CI 0.033-0.058) and AFM (β 0.979, 95%CI 0.667-1.290). No significant association was observed between GFM and MS risk components’ score (β 0.077, 95%CI -0.089-0.243).

**Conclusion:** The results from this study indicated that AG ratio is a stronger predictor of MS than waist circumference and AFM in young Thai adults. The role of AG ratio for the diagnosis of MS needs to be further investigated.

## INTRODUCTION

Metabolic syndrome (MS) refers to a complex metabolic disorder characterized by abdominal obesity, dyslipidemia, increased blood pressure and insulin resistance. Despite the fact that controversy remains around the underlying pathophysiological processes leading to the development of the MS (insulin resistance and/or hyperinsulinemia versus abdominal obesity), there is increased recognition that abdominal obesity is the most prevalent feature of the MS. There is also substantial evidence supporting the notion that an excess of abdominal fat is a predictor of insulin resistance, type 2 diabetes, cardiovascular disease (CVD) and the presence of related metabolic abnormalities commonly associated with the MS (1,2,3,4,5,6,7,8,9,10,11).

The National Cholesterol Education Program (NCEP) -Adult Treatment Panel III (ATP III) accepted abdominal adiposity (assessed by waist circumference) as 1 of 5 criteria for the diagnosis of MS (12). Presence of 3 of the 5 criteria are required for this diagnosis and each component carries equal weight. However, an increased waist circumference does not always mean high-risk abdominal obesity. Waist circumference is determined by several structures of the abdomen and pelvis: bone, muscles, subcutaneous and intraabdominal fat, abdominal viscera, blood vessels and also connective tissue. Using waist circumference as an important parameter for identifying abdominal adiposity status might mislead the diagnosis.

Abdominal adiposity can be assessed by different anthropometric measurements such as waist circumference, waist-to-hip ratio and imaging studies. A number of imaging methods exist for the estimation of abdominal obesity, including computed tomography, magnetic resonance imaging and dual-energy X-ray absorptiometry (DXA). Of these, DXA provides a reliable estimate of body composition. This technique is quick, accurate and exposes subjects to minimal amounts of ionizing radiation (13,14,15,16). Although DXA was originally used to measure bone density and total body composition, recent improvements in the software allow it to determine abdominal fat mass by separation of the body into regions of interest including the android and gynoid regions (17,18,19). The amount of body fat in the android region may confer increased metabolic risk. Up to date, the possible role of measuring abdominal fat composition in association with MS has not been well described. The purpose of this study was to evaluate the association between abdominal adiposity estimated by DXA and MS risk components in young Thai adults.

## METHODS

**Study Population**

This study was carried out within the study investigating the relationship between birth weight and MS, a cohort study using the previous data from Chiang Mai low birth weight study (CMLBWS). For the CMLBWS, the original objective was to study the prevalence and the risk factors of LBW in 2 184 pregnant Thai women from 1989 to 1992. Of the original 2 184 CMLBWS maternal subjects, 770 offspring were interested to participate in the cohort study of the relationship between birth weight and MS. Only 418 offspring of the 770 participants entering the cohort study were interested in this study. Of these 418 participants, 27 participants were excluded due to incomplete biochemical data. Finally, 391 offspring were included in this study. Written informed consent was obtained from all participants and the study was approved by the scientific ethics committee of the Faculty of Medicine, Chiang Mai University.

**Anthropometric Measurement**

The height and weight of each participant were measured while participants were wearing a light robe and no shoes. Body mass index (BMI) was calculated as weight (kg) divided by height squared (m2). Waist circumference was measured at the narrowest point between the lower border of the rib cage and the iliac crest.

**Biochemical Parameters**

Blood pressure was measured twice from the right brachial artery in a sitting position following a 5-minute rest using an automatic machine. The average of these two blood pressure measurements was recorded.

Following fasting for 12 hours, the blood samples (5 mL) were collected for the estimation of fasting plasma glucose (FPG), triglyceride and high-density lipoprotein (HDL) cholesterol levels.

**Regional Body Composition Measurements by DXA**

Body composition was measured by DXA machine (Hologic Discovery A, Hologic Inc., Bedford, MA) equipped with software version 12.3. The machine was calibrated daily using a standard phantom provided by Hologic manufacturer. The in vivo precision of the machine was 2.0%.

**Figure 1:**
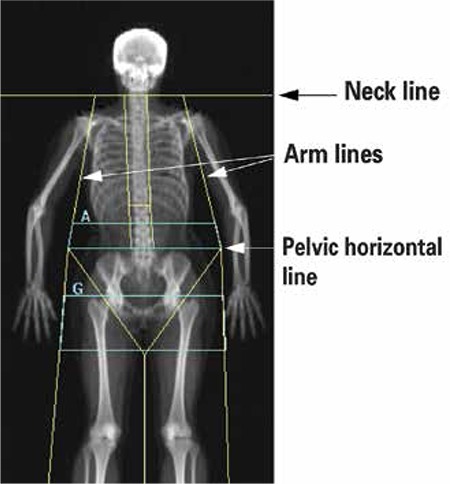
Android and gynoid regions of interest (ROI)

Regional body composition parameters consisting of fat mass (kg), lean mass (kg) and percentage fat (%) were measured in the android and gynoid regions. The regions of interest (ROI) were defined using the software provided by the Hologic manufacture (Figure 1):

Android (A) ROI height is 20% of distance from pelvic horizontal cut line to neck cut line using arm cut lines as lateral boundaries.

Gynoid (G) ROI height is 2 X the height of the android ROI using leg cut lines as lateral boundaries. Upper boundary is below the pelvic horizontal cut line by 1.5 X the height of the android ROI.

Android to gynoid ratio (AG ratio) was determined by using fat percentage in A and in the G regions.

**Definition of Metabolic Syndrome**

MS was defined according to the NCEP-ATP III report criteria (12). Participants were deemed to have MS if they had three or more of the five risk factors: abdominal obesity, elevated triglycerides, reduced HDL cholesterol, elevated blood pressure and elevated FPG listed in Table 1.

Abdominal obesity was defined as waist circumference >90 cm in males and >80 in females based on the cut-off points for Asian population (20).

**Statistical Analysis**

All analyses were conducted using Stata® version 11.0 (StataCorp, Texas, USA). Continuous data are presented as means ± standard deviations (SDs) or as numbers and percentages. All continuous variables in this study showed a normal distribution. Differences were calculated by student’s t-test. Correlation analyses of android fat mass (AFM), gynoid FM (GFM) and AG ratio with waist circumference were performed using Pearson’s correlation. MS risk components including elevated triglycerides, reduced HDL cholesterol, elevated blood pressure and elevated FPG were recoded as 0 (absence of risk) and 1 (presence of risk) using ATP III criteria. Sum scores of MS risk component was generated (MS risk components’ score) and was used for further analysis (0-4). Beta coefficient (β) and 95% CI were applied in linear regression analysis to examine the relationships of waist circumference, AFM, GFM and AG ratio with MS risk components’ score adjusted for gender, BMI and birth weight status. All statistical tests were two-tailed and p<0.05 was considered statistically significant.

## RESULTS

Table 2 shows the basic and anthropometric characteristics of the participants with and without MS. Of the 391 study population (20.4±0.4 years of age), 5.9% (n=23) fulfilled the criteria of MS. 3.3% (n=13) of the participants had obesity, defined by BMI >30 kg/m2. There were no age, birth weight, or gender differences between the two groups. Participants with MS compared to those without MS had higher serum triglycerides, FPG, systolic blood pressure (SBP) and diastolic blood pressure (DBP) levels and lower HDL cholesterol.

**Comparison of Body Composition and Regional Fat Distribution Parameters in Participants with and without Metabolic Syndrome**

The detailed differences in body composition and regional fat depots of the two groups are presented in Table 3. The mean height, weight, BMI and waist circumference were significantly higher in the subjects with MS than in those without MS. Similarly, the regional fat distribution parameters were significantly greater in the participants with MS compared with those without MS (p≤0.001), except for percent gynoid fat (p=0.12). When correlation analyses between body composition parameters were performed, most of them showed significant positive correlation except GFM demonstrating a negative correlation with weight (Table 4).

**Association of Body Composition Parameters with Metabolic Syndrome Risk Components**

The results of correlation analyses between waist circumference, AFM, GFM, AG ratio and the MS risk components are shown in Table 5. Positive correlation was observed of serum triglycerides, SBP, DBP and FPG with body composition parameters. HDL cholesterol was negatively associated with waist circumference, AFM and AG ratio. There were no significant associations of GFM with HDL cholesterol, SPB, DPB and FPG.

Linear regression analyses were performed to examine the association of each body composition parameters (waist circumference, AFM, GFM and AG ratio) with the MS risk components’ score. The univariable analysis showed significant positive association with the MS risk components’ score for all parameters. However, after adjusting for confounding variables, the positive association between GFM and the MS risk components’ score no longer existed. Despite the significant positive association between waist circumference (β=0.046), AFM (β=0.979) and AG ratio (β=1.887), the greatest association was observed with AG ratio.

## DISCUSSION

The relationship between abdominal fat and MS has been extensively explored during the past few years. The results support the evidence that abdominal fat is a major predictor of insulin resistance, CVD and other metabolic abnormalities (21,22). However, most of the studies have been focused the anthropometric measurements such as waist circumference, waist to hip ratio and BMI which are operator-dependent. Those traditional measurements are used to quantify excess weight or size, not the abdominal fat directly. DXA body composition analysis may be superior to anthropometric measurements for evaluating the metabolic risk. Advance DXA technology and software has the ability to accurately identify fat mass and lean mass and their distribution throughout the body with high precision and the ability to quantify AFM and AGM (23,24,25).

There is accumulating evidence that the body fat distribution may be the prognostic indicator for CVD and MS risk (9,10,26,27). However, there are limited studies evaluating the association of DXA-measured AFM, GFM and AG ratio with metabolic risk factors in children and adolescents (28,29). The respective contribution of the AFM, GFM and AG ratio assessed by DXA to cardiovascular risks remains controversial. Aucouturier et al (28) reported that in obese children and adolescents, AF rather than GF was associated with increased insulin resistance and AG fat ratio may be a useful parameter to assess the relationship between fat distribution and insulin resistance. Another study confirmed the positive association between AF and the occurrence of cardio metabolic risk factors (29).

Relationship between basic measured parameters including height, weight and BMI and body composition parameters including waist circumference, AFM, GFM and AG ratio was explored and the result showed significant positive correlation except for GFM which demonstrated a negative correlation with weight as depicted in Table 4. Correlation analysis of waist circumference, AFM, GFM and AG ratio with MS risk components’ score also showed good correlation as seen in Table 5. The greatest correlation was observed between AFM and MS risk components’ score.

MS risk components’ score was used in this study to determine the association with each body composition parameter (waist circumference, AFM, GFM and AG ratio). Linear regression analysis was used to determine the strength of the association expressed by β coefficient. For univariable analysis, significant positive association with the MS risk components’ score was found for all four body composition parameters (Table 6). However, after adjusting for confounding variables, the positive association between GFM and the MS risk components’ score no longer existed. Additionally, the correlation analysis of the MS risk components and GFM also showed no significant associations of GFM with HDL cholesterol, SPB, DPB and FPG. The results from several studies reported no significant association between gynoid fat and MS risk (26,27,28). The findings suggested that gynoid fat might not be an important body composition parameter. From the multivariable linear regression analysis, we found that the AG ratio was the strongest body composition parameter associated with MS risk component’ score (β=1.887). AFM and waist circumference were also significantly associated with MS risk component’ score with β=0.979 and β=0.046, respectively.

In this study, waist circumference, AFM and AG ratio were associated with MS. Of note, AG ratio rather than waist circumference and AFM was the strongest parameter of MS irrespective of birth weight status and gender. This study has several strengths. First, the body composition parameters measured with advanced DXA technology and its software were used. Second, the offspring subjects were recruited from a well-defined large cohort using the previous data from CMLBWS. Third, the appropriate linear regression analysis was adjusted for important factors including BMI, gender and birth weight status and the MS risk components’ score excluding the abdominal obesity criteria was chosen to quantify the true relationship with body composition parameters. This study also had a few limitations. The study subjects were not truly randomly sampled from the original cohort. Furthermore, our study was limited by its cross-sectional nature and thus, more comprehensive studies are required to determine the role of AG ratio in clinical practice including establishing the indices and reference values for predicting the MS- and obesity-related disease.

Despite the aforementioned limitations, this study is valuable in that it indicated that AG ratio is a stronger predictor of MS in young Thai adults than waist circumference and AFM. The role of AG ratio for the diagnosis of MS needs to be further investigated to provide a foun¬dation for implementation in clinical practice.

**Acknowledgements**

We are grateful for the wiling cooperation of all participants. We also would like to thank Dr. Pien Chiowanich and his co-investigators in the 1990 study. This work was supported by joint funding from the Thailand Research Fund and the Commission of Higher Education (MRG 5280229). This research was also funded by the Faculty of Medicine, Chiang Mai University, Chiang Mai, Thailand.

## Figures and Tables

**Table 1:**
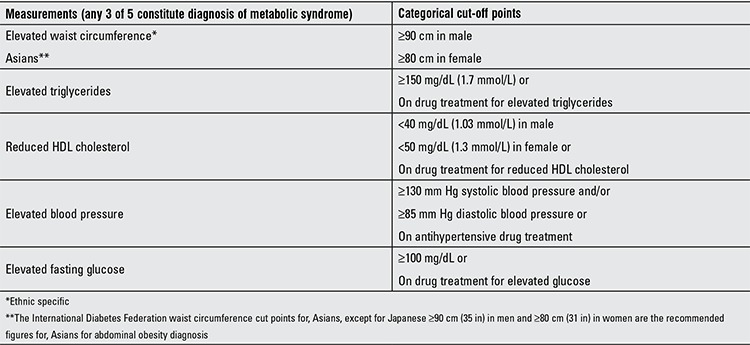
Criteria for clinical diagnosis of metabolic syndrome based on ATP III

**Table 2:**
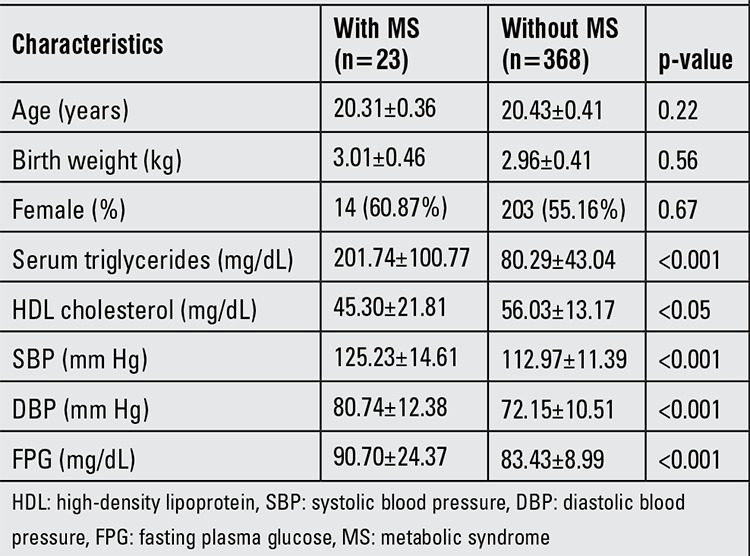
Baseline characteristics of subjects with and without metabolic syndrome

**Table 3:**
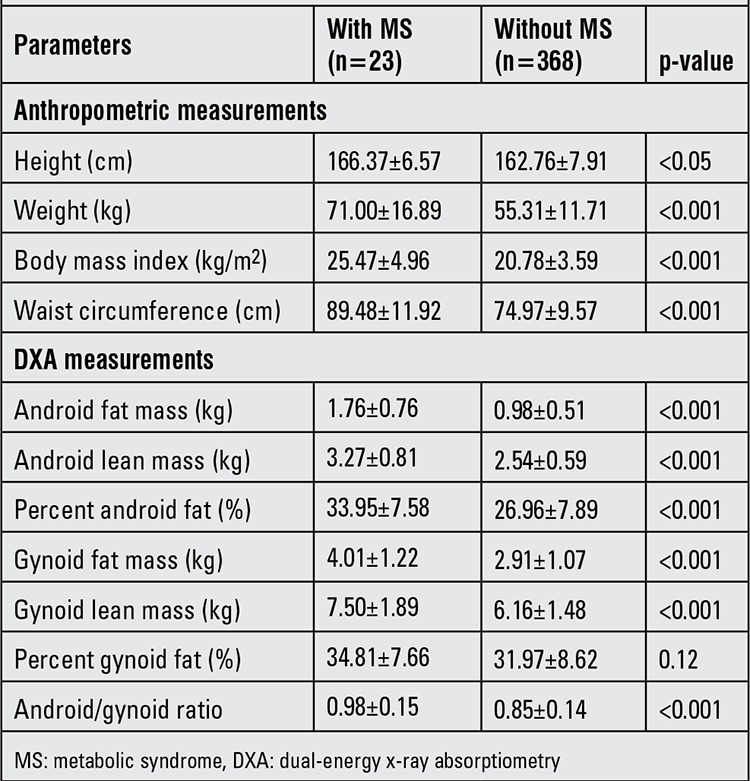
Body composition and regional fat distribution parameters of subjects with and without metabolic syndrome (MS) (mean±SD)

**Table 4:**
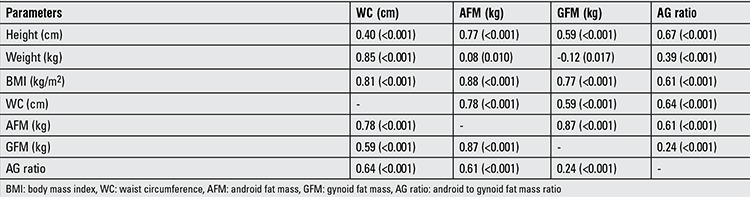
Correlation between height, weight, body mass index and body composition parameters

**Table 5:**
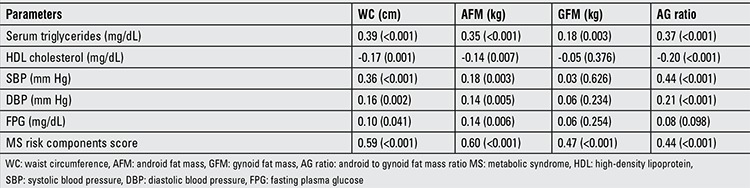
Correlations between WC, AFM, GFM, AG ratio and MS risk components

**Table 6:**
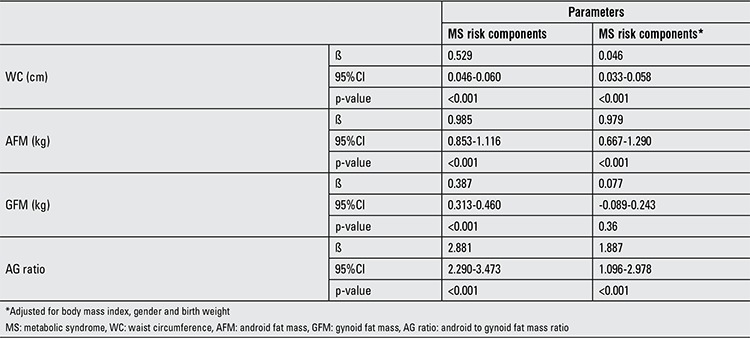
Univariable and multivariable linear regression analyses of waist circumference, android fat mass, gynoid fat mass and android to gynoid ratio against the metabolic syndrome (MS) risk components
